# Evaluation of the clinical and economic impact of delays to surgery in patients with periampullary cancer

**DOI:** 10.1002/bjs5.50161

**Published:** 2019-04-02

**Authors:** R. Pandé, J. Hodson, A. Murray, F. Marcon, M. Kalisvaart, R. Marudanayagam, R. P. Sutcliffe, D. F. Mirza, J. Isaac, K. J. Roberts

**Affiliations:** ^1^ Liver Unit Queen Elizabeth Hospital Birmingham UK; ^2^ Institute of Translational Medicine University Hospitals Birmingham NHS Foundation Trust Birmingham UK; ^3^ Institute of Immunology and Immunotherapy University of Birmingham Birmingham UK

## Abstract

**Background:**

Early treatment is the only potential cure for periampullary cancer. The pathway to surgery is complex and involves multiple procedures across local and specialist hospitals. The aim of this study was to analyse variability within this pathway, and its impact on cost and outcomes.

**Methods:**

Patients undergoing surgery for periampullary cancer (2011–2016) were identified retrospectively and their pathway to surgery was analysed. Patients who had early surgery (shortest quartile, Q1) were compared with those having late surgery (longest quartile, Q4).

**Results:**

A total of 483 patients were included in the study, with 121 and 124 patients in Q1 and Q4 respectively. The median time from initial CT to surgery was 21 days for Q1 *versus* 112 days for Q4 (*P* < 0·001). Diagnostic delays were common in Q4; these patients required significantly more investigations than those in Q1 (endoscopic ultrasonography (EUS): 74·2 *versus* 18·2 per cent respectively, *P* < 0·001; MRI: 33·6 *versus* 20·6 per cent, *P* = 0·036). The median time to diagnostic EUS was 13 days in Q1 *versus* 59 days in Q4 (*P* < 0·001). Some 42·1 per cent of jaundiced patients in Q1 underwent preoperative biliary drainage, compared with all patients in Q4. There were significantly more unplanned admissions and associated longer duration of hospital stay per patient and costs in Q4 than in Q1 (median: 8 *versus* 3 days respectively; €5652 *versus* €2088; both *P* < 0·001). There was a higher likelihood of potentially curative surgery in Q1 (82·6 per cent *versus* 66·9 per cent in Q4; *P* = 0·005).

**Conclusion:**

There is wide variation across the entire pathway, suggesting that multiple strategies are required to enable early surgery. Defining an effective pathway by anticipating the need for investigations and avoiding biliary drainage reduces unplanned admissions and costs and increases resection rates.

## Introduction

The prognosis for patients with periampullary cancer is poor^1^, ^2^. However, for selected patients outcomes have improved; surgical resection with adjuvant chemotherapy offers a 5‐year survival rate of 28 or 40 per cent in patients with pancreatic or periampullary cancer respectively^3^, ^4^. Increasing time to surgery has been shown to relate to unresectability at the time of surgery^5^, as a proportion of patients are found to be unresectable due to occult local or distant spread. Consequently, early surgery is crucially important^6^.

The pathway from presentation, through diagnosis and to treatment for these patients is more complex than that for patients with other tumour types. Centralization has improved surgical outcomes, but made the pathway to surgery complex, requiring clear and rapid communication between local and specialist centres. When cross‐sectional imaging is not diagnostic, obtaining tissue for diagnosis is challenging: brushings from endoscopic retrograde cholangiopancreatography (ERCP) have a low sensitivity for detection of cancer, and, although endoscopic ultrasonography (EUS) has a higher accuracy, it also is often non‐diagnostic and requires repeating^7^, ^8^. The National Institute for Health and Care Excellence (NICE) in the UK has recently recommended PET for patients with potentially resectable pancreatic cancer^9^. In the absence of a coordinated pathway, the need for multiple and/or specialist investigations, as well as multidisciplinary reviews, inevitably prolongs the time from presentation to surgery. Preoperative interventions to correct malnutrition and jaundice, both common problems in patients with periampullary cancer, may also serve to complicate the pathway further^9^, ^10^, ^11^, ^12^. Despite evidence that preoperative biliary drainage is harmful, patients with potentially resectable disease frequently undergo ERCP before referral to a specialist centre^13^.

Thus, the pathway to surgery is complex; although early surgery is clearly desirable, it often may not be achieved. To the authors' knowledge, there are no studies in the literature analysing the pathway and its economic implications for patients with periampullary cancer within the UK. The aim of this study was therefore to define variability within the pathway from presentation to treatment among patients with potentially resectable periampullary cancer, to understand the reasons for this variability, and to compare outcomes between patients undergoing early or late treatment. The hypothesis was that a delay to surgery has a negative impact for patients and healthcare providers.

## Methods

The study was conducted in line with the STROBE guidelines^14^, and was approved by the Institutional Review Board.

All consecutive patients who underwent surgical exploration for potentially resectable periampullary malignancy (pancreatic ductal adenocarcinoma, cholangiocarcinoma and ampullary carcinoma) at University Hospitals Birmingham from January 2011 to December 2016 were reviewed retrospectively. This is a tertiary referral hospital that treats 2·2 million patients per year. Consecutive patients were identified prospectively by a dedicated data manager on a daily basis. Patients who received neoadjuvant chemotherapy were excluded. The primary endpoint was time to surgery, defined as the number of days from the date of initial diagnostic CT to the date of surgery.

The dates of all investigations pertinent to diagnosis and treatment were recorded up to the point of surgery. Signs of biliary and pancreatic duct dilatation with a hypodense mass in the head of the pancreas on CT were considered sufficient for diagnosis. EUS, biopsy, MRI of the liver, staging laparoscopy and PET were used selectively. The lymph node ratio (LNR) was calculated from the ratio of positive lymph nodes from the total lymph node yield. Data related to investigations, interventions, outpatient appointments, clinical tests and operative details were obtained from electronic hospital records. All readmissions, complications and length of stay from hospital episodes, regardless of location (as long as the healthcare provider was a National Health Service (NHS) organization) between initial CT and surgery were identified by review of national data sets including Hospital Episodes Statistics by a dedicated informatics data manager. Costs of treatments/hospital admissions, based on national tariffs, were obtained from the NHS informatics team. The cost of each admission (excluding the admission for the operation) and costs of treatment were totalled for each patient, to provide the cost before surgery.

### Statistical analysis

The time to surgery was summarized using median (i.q.r.) values, and compared across the referring hospitals using the Kruskal–Wallis test. A Jonckheere–Terpstra test was then used, with the hospitals ordered by number of referrals, to assess whether the time to surgery correlated with the number of referrals from a hospital.

The cohort was divided into two groups, based on the quartiles of times from diagnostic imaging to surgery (Q1 and Q4), in order to compare patients with the shortest and longest times to surgery. Patient demographics were then compared between these groups using independent‐samples *t* tests for normally distributed continuous variables and Mann–Whitney *U* tests for non‐normally distributed continuous variables or ordinal variables. Dichotomous variables were assessed with Fisher's exact test. The χ^2^ test was used for other categorical variables. The times between milestones of the patient pathway, and factors relating to treatment and imaging, were then assessed using a similar approach.

A multivariable analysis was performed using a binary logistic regression model, to identify independent predictors of a patient being in Q4 (predictors of longer time to surgery). A backwards stepwise approach was used for variable selection. Continuous variables were divided into categories, before the analysis, to improve model fit. For age and LNR these were based on tertiles of the distribution, whereas for BMI categories of less than 25, 25–29 and 30 kg/m^2^ or above were used, as is conventional.

All analyses were performed using IBM SPSS® version 22 (IBM, Armonk, New York, USA). Two‐sided *P* values were used in all analyses, with *P* < 0·050 deemed to be indicative of statistical significance throughout.

## Results

### Time to surgery

**Figure 1:**
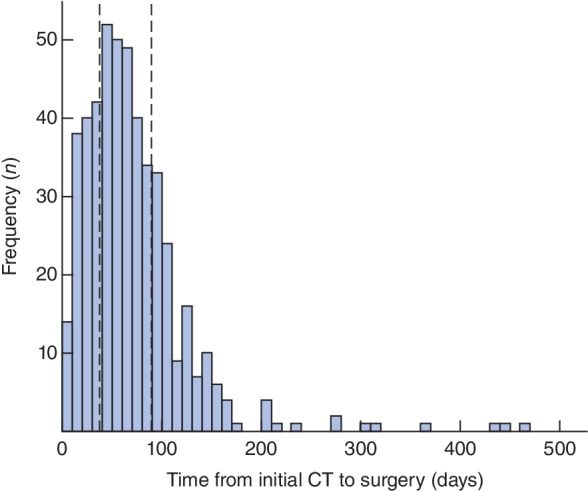
Histogram of the time from diagnostic imaging to surgery. The plot includes all 483 patients who underwent surgery during the study interval. The bars have an interval width of 10 days. The dashed lines represent the lower and upper quartiles (37 and 90 days respectively)

**Figure 2:**
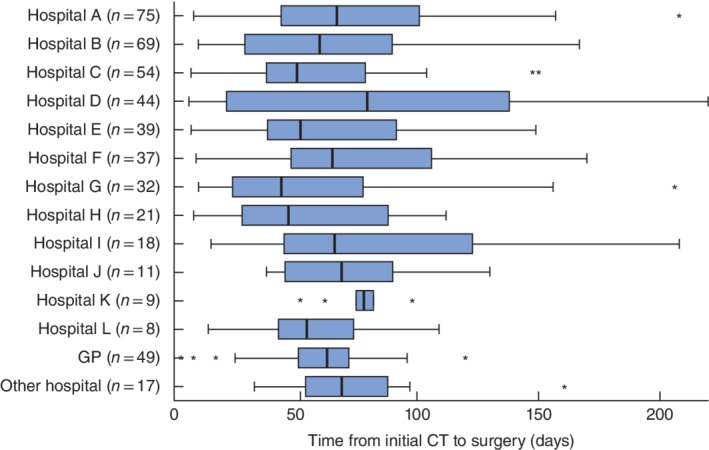
Comparison of time from diagnostic imaging to surgery by the referral hospital. The numbers reported on the *y*‐axis represent the total number of referrals during the period; ‘other hospital’ groups hospitals with fewer than five referrals. Median values, interquartile ranges and ranges are denoted by thick bars, boxes and error bars respectively; asterisks represent outliers, defined as values more than 1·5 times the length of the box. The *x*‐axis was truncated at 220 days as 99 per cent of patients fell within this range. GP, general practitioner. *P* = 0·203 for comparisons across hospitals A–L (Kruskal–Wallis test); *P* = 0·696 when the hospital was treated as an ordinal factor based on total number of referrals (Jonckheere–Terpstra test)

Between 1 January 2011 and 31 December 2016, a total of 483 patients with periampullary cancer had initial diagnostic imaging. The median time from imaging to surgery was 61 (i.q.r. 37–90) days (*Fig*. 1). Comparisons across the referring hospitals are shown in *Fig*. 2. A total of 12 hospitals had at least five referrals during the study period, with a range of 8–75 referrals. Times to surgery were similar across these 12 hospitals (*P* = 0·203), with medians ranging from 44 (i.q.r. 24–78) to 80 (22–138) days. No significant correlation between the number of referrals and time to surgery was detected (*P* = 0·696).

### Patient demographics

Patients in the lower (Q1, 37 days or less) and upper (Q4, at least 90 days) quartiles with regard to time to surgery were identified and interrogated further, giving sample sizes for analysis of 121 and 124 respectively. Patient demographics were generally similar in the two groups for sex, BMI, smoking status and Charlson Co‐morbidity Index (*Table* 1).

**Table 1:** Comparison of patient and diagnostic factors

	Quartile of time from CT to surgery		
	Q1 (*n* = 121)	Q4 (*n* = 124)	*P* †
Age at surgery (years)*	66·2(9·5)	68·5(9·2)	0·056‡
Sex ratio (M : F)	63 : 58	70 : 54	0·523
BMI (kg/m^2^)*	26·2(4·9)	26·0(5·1)	0·786‡
Smoking status			1·000
Non‐smoker	95 (78·5)	97 (78·2)	
Smoker	11 (9·1)	11 (8·9)	
Ex‐smoker	15 (12·4)	16 (12·9)	
Charlson Co‐morbidity Index > 2	41 of 119 (34·5)	52 (41·9)	0·238

Values in parentheses are percentages unless indicated otherwise;

* values are mean(s.d.).

† Fisher's exact test, except

‡ *t* test.

### Patient pathway

The median time to surgery was 112 days in Q4 and 21 days in Q1. Breaking this pathway down into individual components showed that all stages were significantly longer in Q4 than in Q1 (*P* < 0·001) (*Table* 2). The largest difference was in the time from the specialist clinic to surgery: median 75 days for Q4 *versus* 7 days for Q1.

**Table 2:** Components of the patient pathway

		Quartile of time from CT to surgery		
	*n*	Q1 (*n* = 121)	Q4 (*n* = 124)	*P* ‡
Time (days)*				
CT to surgery	245	21 (14–29)	112 (99–142)	< 0·001
CT to referral	222	3 (1–6)	20 (7–41)	< 0·001
CT to final PBD†	157	3 (0–8)	14 (2–46)	< 0·001
Referral to specialist MDT	218	3 (1–7)	4 (2–15)	< 0·001
Specialist clinic to surgery	241	7 (1–14)	75 (45–103)	< 0·001

Values are median (i.q.r.).

* Where pathway components were reversed (for example, where patients were referred before imaging) a time of 0 days was assigned.

† Includes only those patients with jaundice who had preoperative biliary drainage (PBD); see *Table* 3 for further details. MDT, multidisciplinary team.

‡ Mann–Whitney *U* test.

### Diagnostic tests

All patients had at least one diagnostic CT scan, but the total number of scans required was significantly higher in the Q4 group (mean per patient: 1·8 *versus* 1·0 in Q1; *P* < 0·001) (*Table* 3). Patients in Q4 were significantly more likely to require EUS than those in Q1 (74·2 *versus* 18·2 per cent respectively; *P* < 0·001). In addition, where EUS was performed, patients in Q4 had a greater number of tests (mean 1·4 *versus* 1·1 per patient; *P* = 0·024) and had significantly longer time from initial CT to both their first (median 38 *versus* 13 days; *P* < 0·001) and diagnostic (59 *versus* 13 days; *P* < 0·001) EUS. Patients in Q4 were also significantly more likely to require MRI than those in Q1 (33·6 *versus* 20·6 per cent respectively; *P* = 0·036), and tended to have a higher rate of PET, although this was not significantly different (5·9 *versus* 0·9 per cent; *P* = 0·069).

**Table 3:** Diagnostic tests and management of jaundice

		Quartile of time from CT to surgery		
	*n*	Q1 (*n* = 121)	Q4 (*n* = 124)	*P* §
Diagnostic tests				
CT	245	121 (100)	124 (100)	–
Total no. of scans	245	122 (1·0 per patient)	229 (1·8 per patient)	< 0·001¶
EUS	245	22 (18·2)	92 (74·2)	< 0·001
Total no. of EUS scans	114†	24 (1·1 per patient)	133 (1·4 per patient)	0·024¶
Time from CT to first EUS (days)*	114†	13 (7–19)	38 (19–61)	< 0·001¶
Time from CT to diagnostic EUS (days)*	105†	13 (7–19)	59 (39–80)	< 0·001¶
MRI	226‡	22 of 107 (20·6)	40 of 119 (33·6)	0·036
PET	226‡	1 of 107 (0·9)	7 of 119 (5·9)	0·069
Management of jaundice				
No. of patients with jaundice	245	107 (88·4)	112 (90·3)	0·682
ERCP	219	42 of 107 (39·3)	112 of 112 (100)	< 0·001
No. of patients having ERCP	219	29 of 107 (27·1)	84 of 112 (75·0)	< 0·001
Total no. of procedures	154†	46 (1·1 per patient)	142 (1·3 per patient)	0·103¶
PTC	219	6 of 107 (5·6)	23 of 112 (20·5)	0·001
Total no. of procedures	29†	6 (1·0 per patient)	39 (1·7 per patient)	0·058¶
PBD	219	45 of 107 (42·1)	112 of 112 (100)	< 0·001
No. of patients having PBD before referral	216‡	37 of 106 (34·9)	97 of 110 (88·2)	< 0·001
No. of patients with > 1 attempt at PBD	219	6 of 107 (5·6)	41 of 112 (36·6)	< 0·001
Total no. of procedures	157†	51 (1·1 per patient)	181 (1·6 per patient)	0·002¶
Time from CT to final PBD (days)*	157†	3 (0–8)	14 (2–46)	< 0·001¶

Values in parentheses are percentages unless indicated otherwise;

* values are median (i.q.r.).

† In the subgroup of patients for whom at least one procedure was performed.

‡ Excludes patients for whom details of additional imaging or timing of the earliest preoperative biliary drainage (PBD) were not available. EUS, endoscopic ultrasonography; ERCP, endoscopic retrograde cholangiopancreatography; PTC, percutaneous transhepatic cholangiography.

§ Fisher's exact test, except

¶ Mann–Whitney *U* test.

### Treatment of jaundice

Rates of jaundice were similar in the two groups: 88·4 per cent in Q1 and 90·3 per cent in Q4 (*P* = 0·682) (*Table* 3). In those with jaundice, patients in Q4 were significantly more likely to undergo preoperative biliary drainage than those in Q1 (100 *versus* 42·1 per cent; *P* < 0·001). Where biliary drainage was performed, patients in Q4 required significantly more procedures to achieve successful drainage than those in Q1: mean 1·6 *versus* 1·1 procedures per patient (*P* = 0·002). Successful biliary drainage was achieved a median of 3 and 14 days after diagnostic CT in Q1 and Q4 respectively (*P* < 0·001).

### Preoperative admissions and costs of treatment

Patients in Q4 required significantly more hospital admissions, including for performance of initial CT and surgery, than those in Q1 (median 3 *versus* 1 respectively; *P* < 0·001) (*Table* 4). Consequently, the Q4 group also had a significantly longer cumulative length of stay (median 8 *versus* 3 days; *P* < 0·001). In light of this, and the fact that the Q4 group required a greater number of scans and procedures, these patients had a median cost before surgery of €5652, which was significantly greater than the €2088 in Q1 (*P* < 0·001).

**Table 4:** Comparisons of admissions, tumour and operative factors

		Quartile of time from imaging to surgery		
	*n*	Q1 (*n* = 121)	Q4 (*n* = 124)	*P* §
Preoperative admissions				
No. of hospital episodes*	218	1 (1–1)	3 (2–5)	< 0·001¶
Cumulative length of stay (days)*	218	3 (0–8)	8 (1–20)	< 0·001¶
Costs before surgery (€)*	218	2088 (0–3666)	5652 (3299–8450)	< 0·001¶
Tumour factors				
Diagnosis	245			< 0·001
Ductal adenocarcinoma		95 (78·5)	66 (53·2)	
Cholangiocarcinoma		9 (7·4)	25 (20·2)	
Ampullary cancer		17 (14·0)	33 (26·6)	
T category†	182			0·185¶
T0		0 of 100 (0)	3 of 82 (4)	
T1		5 of 100 (5·0)	5 of 82 (6)	
T2		6 of 100 (6·0)	10 of 82 (12)	
T3		86 of 100 (86·0)	59 of 82 (72)	
T4		3 of 100 (3·0)	5 of 82 (6)	
N category†	182			0·031¶
N0		20 of 100 (20·0)	28 of 82 (34)	
N1		78 of 100 (78·0)	53 of 82 (65)	
N2		2 of 100 (2·0)	1 of 82 (1)	
Lymph node ratio*, †	183	0·18 (0·06–0·34)	0·13 (0·00–0·29)	0·062¶
Operative factors				
Operation	245			0·005
Resection		100 (82·6)	83 (66·9)	
Bypass		21 (17·4)	41 (33·1)	
Vascular reconstruction†	182	20 of 99 (20)	10 of 83 (12)	0·163
R1 status†	183	33 of 100 (33·0)	13 of 83 (16)	0·010

Values in parentheses are percentages unless indicated otherwise;

* values are median (i.q.r.). T and N categories were assessed pathologically for resections and radiologically for bypasses.

† Includes only patients who had a resection.

§ Fisher's exact test, except

¶ Mann–Whitney *U* test.

### Tumour and operative factors

Patients with the longest time from imaging to surgery (Q4) had a significantly lower proportion of ductal adenocarcinoma (53·2 per cent *versus* 78·5 per cent for Q1; *P* < 0·001) (*Table* 4). There was no significant difference in T category or LNR between the groups, although N category was significantly higher in the Q1 group (*P* < 0·031). Resection rates were significantly lower in Q4: 66·9 per cent *versus* 82·6 per cent in Q1 (*P* = 0·005). Patients in Q1 had a higher rate of vascular reconstruction (20 *versus* 12 per cent for Q4; *P* = 0·163), and those treated by resection had a significantly higher rate of positive resection margins (33·0 *versus* 16 per cent respectively; *P* = 0·010).

### Predictors of extended times to surgery

Multivariable analysis was performed to identify factors independently associated with patients in Q4 (*Table* 5). The number of CT scans was not included in this analysis because repeat scans were performed to check for progression of disease in those with long times to surgery. Hence, the requirement for multiple CT scans was a result of an extended time to surgery rather than a causal factor.

**Table 5:** Predictors of extended times from CT to surgery

	Univariable analysis		Multivariable analysis	
	Odds ratio	*P*	Odds ratio	*P*
Age at surgery (years)		0·019		0·088
< 65	1·00 (reference)		1·00 (reference)	
65–74	1·00 (reference)	0·574	2·47 (0·94, 6·50)	0·066
≥ 75	1·00 (reference)	0·007	3·26 (1·02, 10·36)	0·046
Female sex	0·84 (0·51, 1·39)	0·491	n.s.	
BMI (kg/m^2^)		0·536	n.s.	
< 25	1·00 (reference)			
25–29	0·80 (0·46, 1·39)	0·424		
≥ 30	1·17 (0·58, 2·39)	0·656		
Smoking status		0·992	n.s.	
Non‐smoker	1·00 (reference)			
Smoker	0·98 (0·41, 2·37)	0·963		
Ex‐smoker	1·04 (0·49, 2·23)	0·910		
Charlson Co‐morbidity Index > 2	1·37 (0·82, 2·31)	0·231	n.s.	
Diagnosis		< 0·001	n.s.	
Ductal adenocarcinoma	1·00 (reference)			
Cholangiocarcinoma	4·00 (1·75, 9·12)	< 0·001		
Ampullary cancer	2·79 (1·44, 5·43)	0·002		
Jaundice	1·22 (0·54, 2·76)	0·631	n.s.	
T3–4 category	0·32 (0·18, 0·56)	< 0·001	n.s.	
N1–2 category	0·34 (0·20, 0·59)	< 0·001	n.s.	
Lymph node ratio		< 0·001		0·033
0	1·00 (reference)		1·00 (reference)	
0·01–0·20	0·31 (0·16, 0·60)	< 0·001	0·50 (0·19, 1·30)	0·154
> 0·20	0·31 (0·16, 0·58)	< 0·001	0·23 (0·08, 0·70)	0·010
No. of EUS scans		< 0·001		< 0·001
0	1·00 (reference)		1·00 (reference)	
1	9·6 (5·0, 18·2)	< 0·001	11·9 (4·7, 30·7)	< 0·001
> 1	46·4 (10·5, 205·1)	< 0·001	67·2 (7·1, 635·8)	< 0·001
MRI	1·96 (1·07, 3·58)	0·029	n.s.	
PET	6·6 (0·8, 54·8)	0·079	9·4 (0·6, 142·6)	0·108
No. of PBD procedures		< 0·001		< 0·001
0	1·00 (reference)		1·00 (reference)	
1	11·5 (5·6, 23·8)	< 0·001	10·8 (4·2, 27·8)	< 0·001
> 1	43·3 (15·1, 123·8)	< 0·001	29·9 (7·6, 118·6)	< 0·001

Values in parentheses are 95 per cent confidence intervals. Results are from binary logistic regression analysis, with Q4 *versus* Q1 as the dependent variable; hence odds ratios greater than 1 represent a greater chance of extended time to surgery. The multivariable model used a backwards stepwise approach to variable selection, with all factors analysed in the univariable analysis considered for inclusion. The final model was based on 221 patients, after exclusions owing to missing data. n.s., Not selected for inclusion by the stepwise procedure; EUS, endoscopic ultrasonography; PBD, preoperative biliary drainage.

As in the univariable analysis, none of the demographic factors was found to be significant in the multivariable model. Of the tumour factors considered, increasing LNR was independently associated with a lower probability of being in Q4 (*P* = 0·033). However, the strongest predictors of a longer time to surgery were the number of times EUS was performed (*P* < 0·001) and preoperative biliary drainage was required (*P* < 0·001).

## Discussion

This retrospective review of the pathway to surgery taken by patients with potentially resectable periampullary cancer identified a broad range in time to surgery for those who undergo early or late surgery (21 *versus* 112 days respectively). Related findings were the particularly long time to establish a tissue diagnosis, where required, for patients in Q4 (13 *versus* 59 days), as well as increased time for every measured stage of the pathway and increased use of every observed investigation. Furthermore, the majority of patients with jaundice in Q1 had surgery without preoperative biliary drainage, whereas draining was required by every jaundiced patient in Q4. Ultimately, patients in Q1 had lower rates of readmission, shorter length of stay, lower treatment costs and a higher resection rate, highlighting the importance of rapid progression from presentation to surgery among these patients.

The start of this pathway was the date of initial CT, which is considered the single best investigation required for diagnosing, or at least raising suspicion of, periampullary cancer; this has been used elsewhere when reviewing outcomes of patients treated within these pathways^10^. The sensitivity and specificity of CT in identifying pancreatic cancer is 70–100 per cent^11^, and its ability to evaluate resectability based on locoregional, distant and vascular involvement is 81–99 per cent^12^, ^13^, ^15^. For some patients, CT can be the single best imaging required to determine definitive management, as CT has been shown to be superior to MRI and ultrasound imaging^12^.

When there is diagnostic uncertainty, ERCP has previously been relied on to provide tissue for diagnosis^16^. However, the role of ERCP in the pathway has devolved from diagnostic to therapeutic, certainly within the era of improved CT accuracy and EUS. However, when EUS is required, this study suggests it is associated with significant delay among patients who proceed slowly to surgery and is, therefore, a particular area of the pathway that is prone to causing delay. Anticipating the need for EUS, rapid reporting of pathology and, when needed, repeating EUS urgently could all reduce time to diagnosis. Same‐day reporting of histological material from suspected cancer is possible^17^, ^18^, ^19^, and is something this study suggests could have a major impact on the cancer pathway.

It is common for patients (42–79 per cent) to have had preoperative biliary drainage before referral to specialist surgical review^20^, ^21^, ^22^, ^23^. However, this is associated with significant harm^24^, including a 4 per cent risk of pancreatitis, haemorrhage and bowel perforation^25^, ^26^, ^27^, ^28^, ^29^, ^30^, ^31^, ^32^, ^33^, ^34^. Biliary colonization following ERCP has been reported to be as high as 27 per cent^35^, ^36^, with a resultant impact on surgical‐site infections after pylorus‐preserving pancreaticoduodenectomy (PPPD)^37^.

A direct‐to‐surgery approach among jaundiced patients, rather than preoperative biliary drainage, is associated with a marked reduction in complications and readmissions^38^. After preoperative biliary drainage, the need for further procedures and stent changes is common due to cholangitis^39^.

In August 2015, a pathway to offer surgery without preoperative biliary drainage was implemented at the authors' centre; before this, surgery without preoperative biliary drainage was performed on an *ad hoc* basis^40^. It is interesting to observe that this reduced the time from CT to referral, suggesting that changes in specialist care pathways influence the behaviour of referring colleagues in non‐specialist centres. Patients within this ‘fast track’ pathway were within the shortest quartile in time to surgery where diagnostic and staging investigations were prioritized and organized in parallel, rather than in series, reducing times to diagnosis and treatment^40^. Patients in the longest quartile to surgery were treated within pre‐existing pathways provided within NHS standard treatment times for cancer. These state that treatment for cancer should be provided within 62 days of referral. The experience of these patients shows that provision of early treatment is a challenge within a nationally funded healthcare system of limited resource without some form of support over and above those provided within existing pathways.

Owing to the multiple benefits, avoidance of preoperative biliary drainage among jaundiced patients is recommended in best practice guidelines^41^ and by NICE^9^, where possible.

The recently published NICE guidelines on pancreatic cancer^9^ aim to ensure quicker and more accurate staging. These guidelines recommend PET for all patients to avoid overtreatment of those with occult metastatic disease. This could potentially delay treatment further, and thus it is important that, where implemented, PET is done with minimal impact to the patient pathway. PET–CT showed low accuracy in differentiating benign from malignant periampullary lesions in 57 per cent of patients^42^, although it had a relatively good ability to detect lymph node metastasis^43^. The recent PET‐PANC study^44^ showed similar sensitivity and specificity between multidetector CT and PET–CT. PET–CT upstaged disease, preventing resection in 11 per cent of patients.

The present study, and others^5^, ^45^, ^46^, however, highlight that treatment is time critical. Consequently, concerns over understaging must be balanced against introducing undue delays to surgery. Indeed, overuse of preoperative investigations prolongs time to surgery and results in complications incurred as a result of them^39^, ^47^.

Patients undergoing resection in both quartiles had similar pathological profiles and yet there was a higher rate of resection, despite more borderline cases requiring vascular reconstruction and involved margins, in the faster quartile.

There are clear limitations to this study. The data were derived from a single hospital and its network of referring teams, and thus may not be generalizable. However, the median time to surgery (61 days) is representative for patients in the UK^40^. There is currently a prospective national audit of pancreatic cancer in the UK, the RICOCHET study, which will map the patient pathway in detail. Aspects of the pathway, such as local preference for use of various diagnostic tests and the surgical approach to borderline resectable and locally advanced cancers, may not be generalizable. The strength of this study is that it originates from a centre that currently performs roughly 10 per cent of the UK pancreatic cancer resections per year (based on hospital resection volume and data on cancer resections available at the UK NHS Hospital Episodes Statistics database)^48^, and it is possible that the data presented here may actually underestimate diagnostic delays in other units, particularly if surgery without preoperative biliary drainage is not routine.

Focusing on establishing rapid diagnosis and staging (including efficient and effective use of EUS) and avoiding preoperative biliary drainage appear to be strategies that can effectively reduce time to surgery. The need for multiple staging investigations must be balanced against risks of cancer progression if they cause delays to treatment. Cost saving from providing early surgery and avoiding preoperative biliary drainage could be redirected into supporting these pathways.

## Disclosure

The authors declare no conflict of interest.
